# Preparation of *Tragopogon graminifolius*-loaded electrospun nanofibers and evaluating its wound healing activity in a rat model of skin scar

**DOI:** 10.3389/fphar.2025.1533010

**Published:** 2025-01-31

**Authors:** Leila Almasi, Elham Arkan, Mohammad Hosein Farzaei, Amin Iranpanah, Cyrus Jalili, Fatemeh Abbaszadeh, Faranak Aghaz, Sajad Fakhri, Javier Echeverría

**Affiliations:** ^1^ Student Research Committee, Kermanshah University of Medical Sciences, Kermanshah, Iran; ^2^ Nano Drug Delivery Research Center, Health Technology Institute, Kermanshah University of Medical Sciences, Kermanshah, Iran; ^3^ Pharmaceutical Sciences Research Center, Health Institute, Kermanshah University of Medical Sciences, Kermanshah, Iran; ^4^ Medical Biology Research Center, Health Technology Institute, Kermanshah University of Medical Sciences, Kermanshah, Iran; ^5^ Neurobiology Research Center, Institute of Neuroscience and Cognition, Shahid Beheshti University of Medical Sciences, Tehran, Iran; ^6^ Departamento de Ciencias del Ambiente, Facultad de Química y Biología, Universidad de Santiago de Chile, Santiago, Chile

**Keywords:** wound, skin scar, *Tragopogon graminifolius* DC., nanofibers, antioxidant, electrospinning

## Abstract

**Background:**

Growing reports are dedicated to providing novel agents for wound healing with fewer adverse effects and higher efficacy. The efficacy of nanofibers composed of polyvinyl alcohol (PVA)/polyethylene oxide (PEO)/chitosan (CS) in promoting wound healing can be attributed to their ability to stimulate collagen production. Among the herbal agents with fewer adverse effects, *Tragopogon graminifolius* DC. [Asteraceae] (*TG*), also called “Sheng” in traditional Iranian medicine, is one of the most efficacious plants for treating various skin injuries due to its several pharmacological and biological effects like anti-inflammatory and antioxidant properties.

**Purpose:**

In the present study, our objective was to assess the wound-healing activity of PVA/PEO/CS nanofibers containing *TG* in a rat model of excision wound repair.

**Methods:**

Synthesized nanofibers from PVA, PEO, and CS were done by the electrospinning method and confirmed by scanning electron microscopy (SEM) and Fourier-transform infrared spectroscopy (FT-IR). The release tests of nanofibers were assessed through the UV-visible method at different time intervals, which were conducted for about 60 h. To evaluate the wound healing effects, rats were divided into four distinct groups, including negative control (untreated), phenytoin cream (as positive control), polymer (PVA/PEO/CS), and drug (nanofiber-containing 50% of *TG* extract; named PVA/PEO/CS/*TG*) groups. All treatments were administered topically once daily for 14 days. Wound size changes were investigated in different time intervals. On the 15th day, nitrite and catalase serum levels were measured. Furthermore, samples of skin tissue were extracted and subjected to histopathological analysis.

**Results:**

PVA/PEO/CS nanofibers containing 1.2 g of PVA, 0.3 g of PEO, and 0.8 g of CS, along with 50% of *TG* extract (PVA/PEO/CS/*TG*) at 17 kV were selected based on its favorable morphology and uniform quality. PVA/PEO/CS/*TG* represented a notable reduction in wound sizes. Moreover, in histopathological analysis, PVA/PEO/CS/*TG* showed a lower presence of inflammatory cells, higher density of dermis collagen fibers, and better regeneration of the epidemic layer. In addition, PVA/PEO/CS/*TG* elevated plasma antioxidant capacity via increasing catalase while reducing nitrite levels.

**Conclusion:**

PVA/PEO/CS/*TG* is a promising wound dressing nanofiber with antioxidant and tissue regeneration potential. These results encourage further studies for the development of *TG* nanofibers as promising agents in treating and accelerating the process of excision wound repair.

## 1 Introduction

The skin, being the largest organ in the human body, comprises approximately 16% of the total body weight and covers approximately 1.5–2 m^2^ of body surface area in adults. It serves as a crucial interface between indoor and outdoor environments, acting as a barrier to safeguard the body against various environmental stressors. Furthermore, it plays a vital role in maintaining homeostasis by preventing water loss and regulating electrolyte levels (Roger et al., 2019). The skin is also a vital part of the body, which protects the body against foreign agents including the invasion of microorganisms, chemical/radiological damage, and dehydration (Naseri-Nosar et al., 2018). Wound is one of the major factors that disrupt the skin’s protective function and exposes the body to protein/water loss, and infection (Ehterami et al., 2018). Wounds are caused by scratching, surgery, abrasion, chemicals, cold, heat, pressure, and shear, or as a result of diseases such as carcinomas or foot injuries (Enoch and Price, 2004; Rappl et al., 2007).

Wound healing is a dynamic and complicated interaction process between cells and mediators. This process entails a series of events, different tissues, and cells including platelets, several growth factors, including fibroblast growth factor (FGF), platelet-derived growth factor (PDGF), transforming growth factor-β (TGF-β), vascular endothelial growth factor (VEGF), and adhesion molecules. In line, inflammatory cells and mediators are also involved in the wound healing procedure including the engagement of polymorphonuclear leukocyte (PMN), macrophages, interferons (IFNs), interleukins (ILs), and tumor necrosis factor (TNF)-α. Additionally, oxidative stress mediators play critical roles in wound regeneration, such as reactive oxygen species (ROS), glutathione peroxidase (GPx), nitric oxide (NO), superoxide dismutase (SOD), and catalase (CAT) (Henry and Garner, 2003; Eming et al., 2007; Bayrami et al., 2018; Shaygan et al., 2021; Wang et al., 2023).

Augmenting evidence highlights the importance of targeting oxidative stress to facilitate the wound-healing processes (Henry and Garner, 2003; Eming et al., 2007; Cano Sanchez et al., 2018; Wang et al., 2023). Several reports underscore the participation of ROS as pivotal regulators throughout different stages of the wound healing process. It is indeed undeniable that ROS plays a role in almost all wound healing processes through its influence on cell proliferation, inflammation, and granulation. ROS presence in low quantities is necessary to combat invasive microorganisms and facilitate cellular survival signaling. However, excessive production of ROS or a compromised ability to detoxify them results in oxidative damage that has an adverse impact on the process of wound healing in a majority of pathological wounds. Consequently, a delicate equilibrium between ROS generation and scavenging becomes imperative to ensure a prompt and effective wound-healing response (Shaygan et al., 2021; Wang et al., 2023).

So far, multiple approaches have been employed for wound healing, including homeopathy, herbal and chemical medicines, and physical methods like laser therapy. Rapid treatment with the least complication and at the same time the lowest cost is the general goal of all treatment methods (Reinke and Sorg, 2012; Pereira and Bártolo, 2016). The ideal dressing serves as a crucial determinant in the process of wound healing. A suitable dressing must not only have sufficient oxygen permeability but also have biological and structural properties similar to the extracellular matrix (Venugopal et al., 2008; Selig et al., 2012). Electrospinning nanofibers have garnered considerable attention in recent years because of their unique properties, including excellent strength, high porosity, and a remarkably large surface area (Wang et al., 2009; Bhardwaj and Kundu, 2010; Abdoli et al., 2020). These special properties have made nanofibers attractive and important, especially in biomedicine. Since nanofiber scaffolds exhibit behavior similar to the extracellular matrix, they are important candidates in functional wound dressing materials and tissue engineering (Liang et al., 2007; Sill and von Recum, 2008; Nematpour et al., 2020).

These nanofibers are superior to wound healing possessing a higher surface-to-volume ratio and having small pores compared to gas, sponge, and hydrogel. Also, when nanofiber scaffolds are used to treat wounds without the use of hemostatic agents, the homeostasis step is performed and fluid accumulation and oxygen infiltration at the wound site are facilitated (Chong et al., 2007). So far, various nanofibers including poly-ethylene oxide (PEO), chitosan (CS), polyvinyl alcohol (PVA), gelatin, fibrinogen, collagen, polycaprolactone (PCL), polyurethane and poly-lactic acid have been evaluated for skin and wound healing (Khil et al., 2003; Choi et al., 2008; Powell et al., 2008). Amongst the aforementioned polymers, PVA, PEO and CS have shown promising advantages towards wound-healing potentials, including cost-effectiveness, continuous and uniform nanofibers with controllable pore structure, high porosity, swelling capacity, cellular adhesion, and the capability to provide warmth and moisture environment to speed up wound healing (Tang et al., 2019; Alven and Aderibigbe, 2021; Yuan et al., 2023).

Traditional and ethnomedicine, which are based on long-standing practices, offer cost-effective, straightforward, and effective alternatives for various categories of wounds. These remedies exhibit a diverse array of therapeutic effects that stimulate the healing process (Pereira and Bártolo, 2016). Moreover, the plant kingdom has been a promising source of alternative therapies with fewer side effects and lower economic costs, which are analyzed and applied to treat skin disorders (Reuter et al., 2010; Tabassum and Hamdani, 2014; Shedoeva et al., 2019; Majtan et al., 2021). *Tragopogon graminifolius* DC. [Asteraceae] (*TG*), is locally known as the “Sheng” or “Lahiat-o-tis” in traditional Iranian medicine (TIM) (Farzaei et al., 2014a). It is one of the plants widely growing in the Western parts of Iran (Mozaffarian, 1996). This plant germinates in spring and summer and belongs to the category of biennial or perennial plants, possessing narrow, linear, and bayonet leaves. So far, about 127 species of this genus have been registered in the online Encyclopedia of Life, and there are about 25 species of *TG* in Iran (Krzaczek and Smolarz, 2014). The identified compounds in the *Tragopogon* genus are phenolic compounds, terpenes, saponins, benzylphtalides, coumarins, bibenzyls, and sterols with confirmed potential effects in wound-healing (Sareedenchai et al., 2009; Farzaei et al., 2014a; Abdalla and Zidorn, 2020). In the TIM, *TG* has been utilized to treat wounds, different gastrointestinal ailments, pulmonary infections, hemorrhage, and hepatic disorders (Farzaei et al., 2014a; 2014b; Krzaczek and Smolarz, 2014). Moreover, several studies have confirmed the pharmacological and biological properties of *TG* including anti-inflammatory, antioxidant, analgesic, antitumor, antimicrobial, and hepatoprotective properties, in addition to its beneficial effects in gastrointestinal disorders (Abdalla and Zidorn, 2020).

Hence, the objective of the present study was to evaluate the efficacy of the PVA/PEO/CS nanofibers containing *TG* extract in promoting wound-healing in a rat model of excision wounds employing antioxidative effects.

## 2 Materials and methods

### 2.1 Chemicals

Ethanol, PVA (MW_average_ 72,000 g/mol, 98% hydrolyzed), CS (MW 270,000 g/mol, 85% degree of deacetylation), PEO, tween 80 (polysorbate 80), potassium bromide (KBr), ammonium molybdate, *N*-(1-naphthyl) ethylenediamine dihydrochloride (NED), hydrogen peroxide (H_2_O_2_), sulfanilamide, phosphoric acid (H_3_PO_4_), xylazine, and acetic acid were purchased from Merck Company (Germany). Ketamine and phenytoin cream were procured from Alfasan (Woerden, Netherlands) and Behvazan Company (Iran), respectively. All used compounds and chemicals were of analytical grade.

### 2.2 Plant material and preparation of the *TG* extract

The *T. graminifolius* DC. [Asteraceae] was collected in April 2020 from around the Kermanshah province, Iran. The plant name has been checked with the World Flora Online (WFO) site (www.worldfloraonline.org) and approved by the Central Herbarium of Tehran University, School of Biology, College of Science, University of Tehran, Tehran, Iran. The plant voucher number (43603) was stored at the University of Tehran in the Central Department of Botany. The aerial parts of *TG* were washed and dried in the shadow under suitable conditions. The dried *TG* was powdered by a mechanical grinder. Then, 100 mg of the dried plant was poured into an Erlenmeyer flask and extracted with 70% ethanol. The resulting mixture was placed in a shaker for about 60 h. In the next step, the mixture is filtered and the resulting solution is concentrated under vacuum by a rotary apparatus (Heidolph Instruments GmbH and Co., Germany) to obtain the desired extract. Three extracts with concentrations of 10, 50, and 90% w/v *TG* were prepared and tested to obtain the most effective extract (Farzaei et al., 2014b).

### 2.3 Preparation of polymeric solution

To prepare the polymer solution, 0.3 g of CS was dissolved in 10 mL of acetic acid. In the second beaker, 0.2 g of PEO was dissolved in 10 mL of acetic acid. Also, in the third beaker to prepare a PVA solution, 0.6 g of the polymer was added to 4.4 mL of distilled water. In order to prepare the working solution, the ratios of each polymer were chosen in such a way that the final product consisted of 1:1:0.3 g for CS, PVA, and PEO, respectively. The *TG* extract was then added as 10, 50, and 90% w/v into the final working solution.

### 2.4 Preparation of nanofibers by electrospinning method

After preparing polymer solutions containing *TG* extracts with concentrations of 10, 50, and 90% (w/v), the desired experiments were performed to achieve the proper nanofibers with an electrospinning device. To attain the best nanofibers, different parameters of the electrospinning such as nozzle-to-collector distance, voltage, and material concentration were thoroughly examined. Notably, distances of 8, 12, and 17 cm and voltages of 8, 12, and 17 kV were investigated and a light microscope was used to find the best fiber. The best nanofibers were obtained at a distance of 12 cm from the nozzle at an injection rate of 0.7 mL per hour, and the working voltage of 17 kV with 50% *TG* extract. The study was done on room temperature and humidity.

### 2.5 Scanning electron microscope (SEM) analysis

The main characteristics of nanofibers including morphology, diameter distribution, and shape were evaluated utilizing a Scanning Electron Microscopy (SEM) (FEI Model Quanta 450 FEG, Hillsboro, OR, United States), at an operating voltage of 25 kV. All the samples were fixed to an aluminum stub and sputter coated with gold under an argon atmosphere.

### 2.6 Release test

To determine the amount of *TG* extract released from nanofiber, 5 mg of nanofiber was accurately weighed and placed inside the dialysis membrane (Cut-off ∼ 12 kDa), containing 2 mL of phosphate buffer plus 1% v/v Tween 80. The dialysis membrane was then immersed in 50 mL of phosphate buffer (release medium) at pH 7.4 and was stirred at 100 rpm. This process was repeated 3 times and sampling was done at different times (15, 30, 45, 60, 75, 90, 105,120, 150, 180, 210, 240, 300, 360, 420, 1680, 3120, 3360, and 3600 min). The absorbance of the samples was measured by UV-visible apparatus at a wavelength of 263 nm.

### 2.7 Swelling test

To evaluate the swelling of nanofiber, 10 mg of dried samples were immersed in aqueous media containing phosphate buffered saline (PBS). Samples were incubated for 30 min at 37°C. After that, the samples were expelled and then their weights were again accurately measured. The following formula was used to calculate weight changes:

$$\left.Q_{s}\;\left(\%\right)=\left(W_{t}\;–\;W_{0}\right)\;/\;W_{0}\times \;100\right)$$

Q_s_ = Inflation ratio.

W_t_ = Weight of swollen nanofibers.

W_0_ = Weight of primary dry nanofibers.

### 2.8 Mechanical properties of nanofibers

To assess the mechanical properties of the PVA/PEO/CS and PVA/PEO/CS/*TG*, a mechanical tensile testing device was employed (STM-1 DBBP-100, South Korea). The nanofibers were cut to 40 mm × 10 mm and related mechanical properties were tested at room temperature. The tensioning speed was 2 mm/min.

### 2.9 Animals and ethical considerations

In this research, twenty-four male Wistar rats weighing 220 ± 20 g were selected from the Animal House Unit of the School of Pharmacy, Kermanshah University of Medical Sciences (Kermanshah, Iran), and divided into 4 groups of 6 each. Rats were placed in plastic cages under standard laboratory conditions (25°C, 60% humidity, 12-h/12-h dark cycle) and fed with water and chow. All animal procedures were approved by the animal ethics committee of Kermanshah University of Medical Sciences, Iran (IR.KUMS.REC.1398.673) and were performed in adherence to pertinent guidelines and regulations.

### 2.10 Creation of excision wound

The rats underwent anesthesia through intraperitoneal (i.p.) injections of a combination of ketamine and xylazine (80 mg/kg and 10 mg/kg). Subsequently, the back surface of the rats was shaved and sterilized. The excision wound (20 mm × 20 mm) was made on the back through the removal of a section of skin.

### 2.11 Treatment protocol

After 1 week of acclimatization, 24 adult male Wistar albino rats were randomly divided into 4 groups (*n* = 6). The four studied groups included a negative control (untreated), phenytoin cream (as positive control), polymer (PVA/PEO/CS), and drug (nanofiber-containing 50% of *TG* extract; named PVA/PEO/CS/*TG*) groups. All treatments were administered topically once daily for 14 days. The initiation of wound creation was regarded as zero, and the commencement of the treatment occurred 24 h after the creation of the wound. The rats were then sacrificed on the 15th day. Prior to the sacrifice on the 15th day, blood samples were taken from rats in order to evaluate the nitrite and CAT serum levels, which serve as biomarkers for the measurement of oxidative stress.

### 2.12 Wound contraction rate

Wound contraction was evaluated through the utilization of a digital camera to capture photographs. Subsequently, the obtained images were subjected to analysis via ImageJ software to measure the size of the wound area. The wound closure rate was then represented as the percentage of reduction in the zero-day wound size. This rate was computed using the following formula:

Wound contraction (%) = [wound size of the induction day − wound size of the specific day (days 0, 3, 7, 10, and 14) after treatment)]/wound size of the induction day × 100.

### 2.13 Histopathological analysis

For histopathological evaluations, on day 15, the rats were anesthetized through i.p. administration of thiopental sodium, and then skin tissue samples from the wound areas were collected and subjected to fixation using a 10% formalin. After preparing tissues, sections measuring 7 μm in thickness were stained with hematoxylin and eosin (H&E). The Olympus CX23 light microscope, Dino-Lite camera, and DinoCapture 2.0 software were used for visualization of the samples. Histopathological investigations were done by a blinded experimenter.

### 2.14 Nitrite assay

Nitric oxide was measured indirectly because it is unstable. For this reason, we measured its stable metabolites, namely the nitrate and nitrite anions. Nitrite measurement was performed by Griess reaction Griess 1858 (Sun et al., 2003). In this method, deproteinization is necessary and the use of zinc sulfate is the best method compared to other methods. In the Griess reaction, nitrite reacts with sulfanilamide to give an unstable diazonium salt. Then, this salt combines with NED to form purple-colored aromatic compounds that can be used to measure nitrite concentration. This reaction creates a spectrum of purple paint that can be used to measure nitrite concentration. 100 μL of serum was mixed with 50 μL sulfanilamide (dissolved in 5% H_3_PO_4_). Following 5 min keeping of the prepared sample at room temperature, 50 μL of NED solution (0.1% in water) was added to each sample. A purple complex was then formed, the absorption of which was read at 540 nm with a 630 nm reference filter by an ELISA reader. Finally, the standard curve was drawn and results were reported as a percentage (%) of control (Kumar et al., 2009; Fakhri et al., 2022).

### 2.15 Catalase changes

Catalase activity was measured to assess the antioxidant level according to the method of Aebi (1984). Briefly, 20 µL of serum samples were combined via 100 µL of H_2_O_2_ (65 mM) into 96-well plate wells. Thereupon, incubated at room temperature for 4 min. The reaction was stopped through the addition of 100 µL ammonium molybdate (32.4 mM) and a yellow molybdate and H_2_O_2_ complex were formed. The absorbance of the sample was subsequently determined at a wavelength of 405 nm using an ELISA reader (Fakhri et al., 2022). Results were reported as a percentage of control (%).

### 2.16 Statistical analysis

All data were expressed as mean values ± standard deviation (S.D.). Repeated measures of one-way and two-way analysis of variance (ANOVA) and Tukey’s post-hoc analysis were done. The statistical significance was determined by accepting the *p*-values of ∗*p* < 0.05, ∗∗*p* < 0.01, and ∗∗∗*p* < 0.001 with different symbols.

## 3 Results

### 3.1 Morphological studies

**FIGURE 1:**
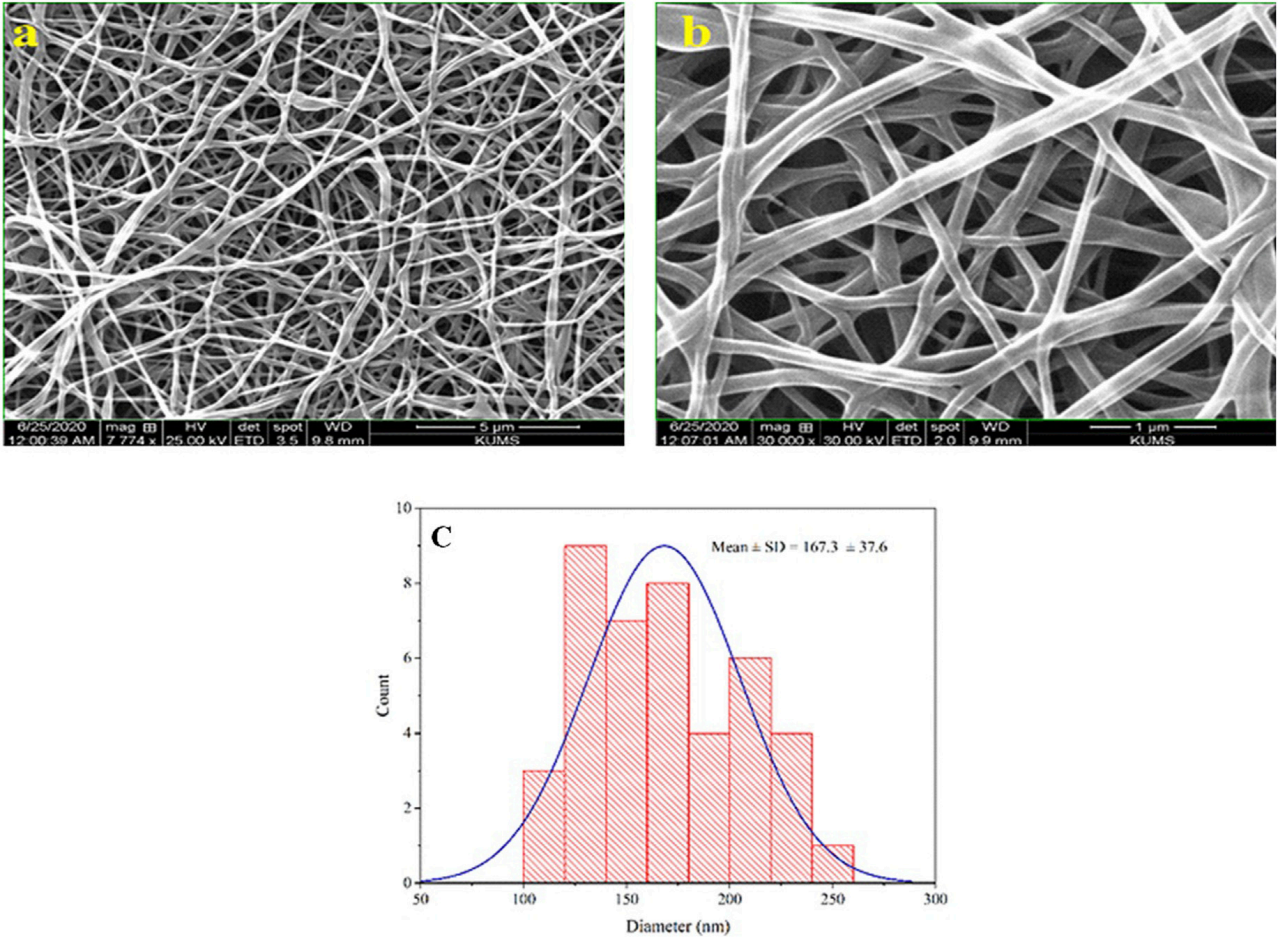
Morphological studies of PVA/PEO/CS nanofibers. SEM images of optimal nanofiber with different magnifications **(A, B)**, and the particle size distribution histogram of optimal nanofiber in 1 µm scale **(C)**.

**FIGURE 2:**
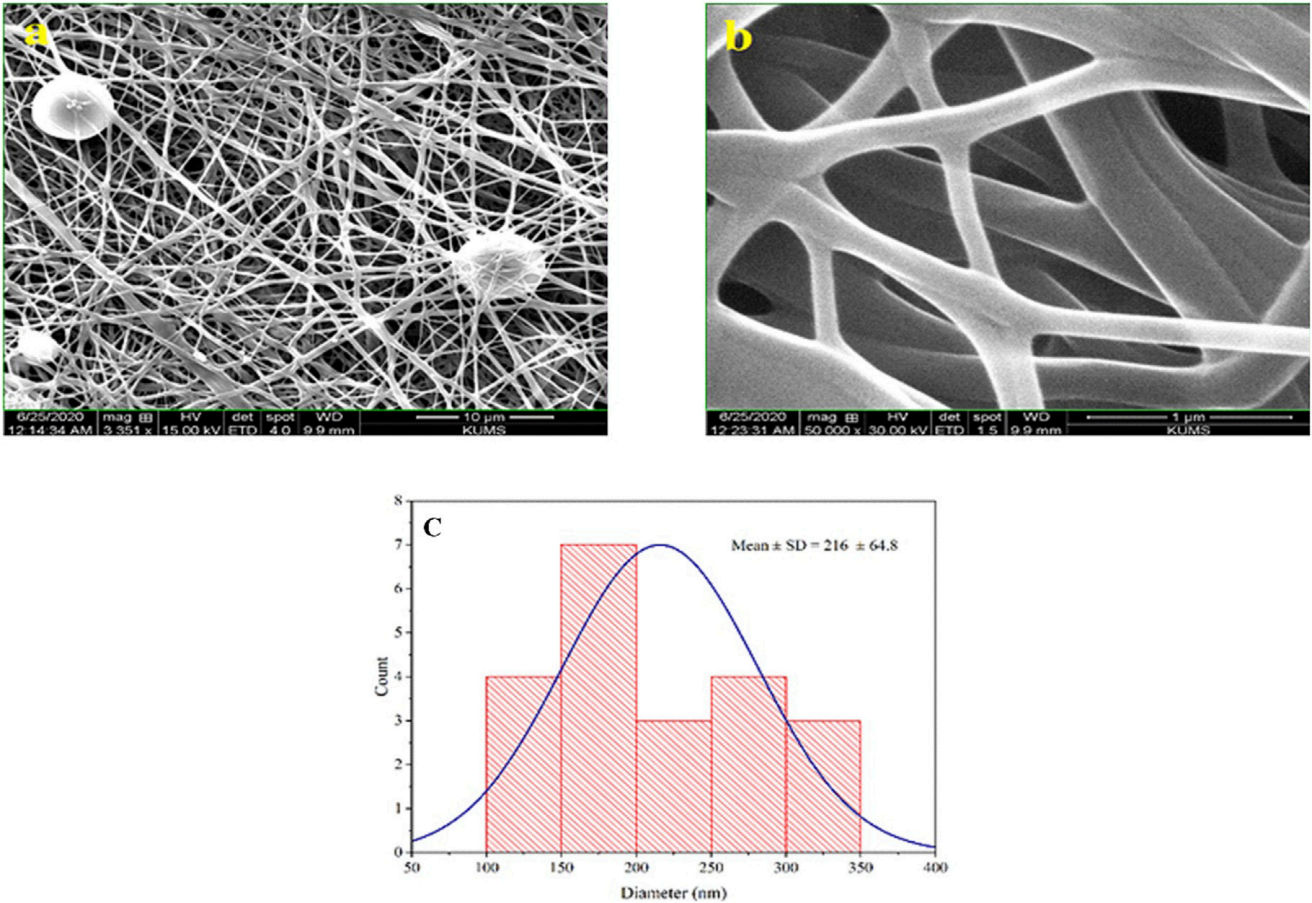
Morphological studies of PVA/PEO/CS/*TG* nanofibers. SEM images of the PVA/PEO/CS nanofiber with 50% *TG* **(A, B)**, and optimal nanofiber particle size distribution diagrams containing 50% *TG* extract in 1 µm scale **(C)**.

After preparing the optimized nanofibers, their morphology and diameter were analyzed through SEM imaging. SEM images related to optimal nanofibers are shown in Figures 1A, B with different magnifications. SEM images of electrospun nanofibers with 50% *TG* are illustrated in Figures 2A, B. Comparing the initial fiber (Figure 1C) and after adding the *TG* extract to the polymeric solution the mean fiber diameter was increased but the overall structure of the nanofiber did not change (Figure 2C). These results confirm that the utilized nanofiber in this study is the suitable system of choice for successfully loading the *TG* extract.

### 3.2 Release test

**FIGURE 3:**
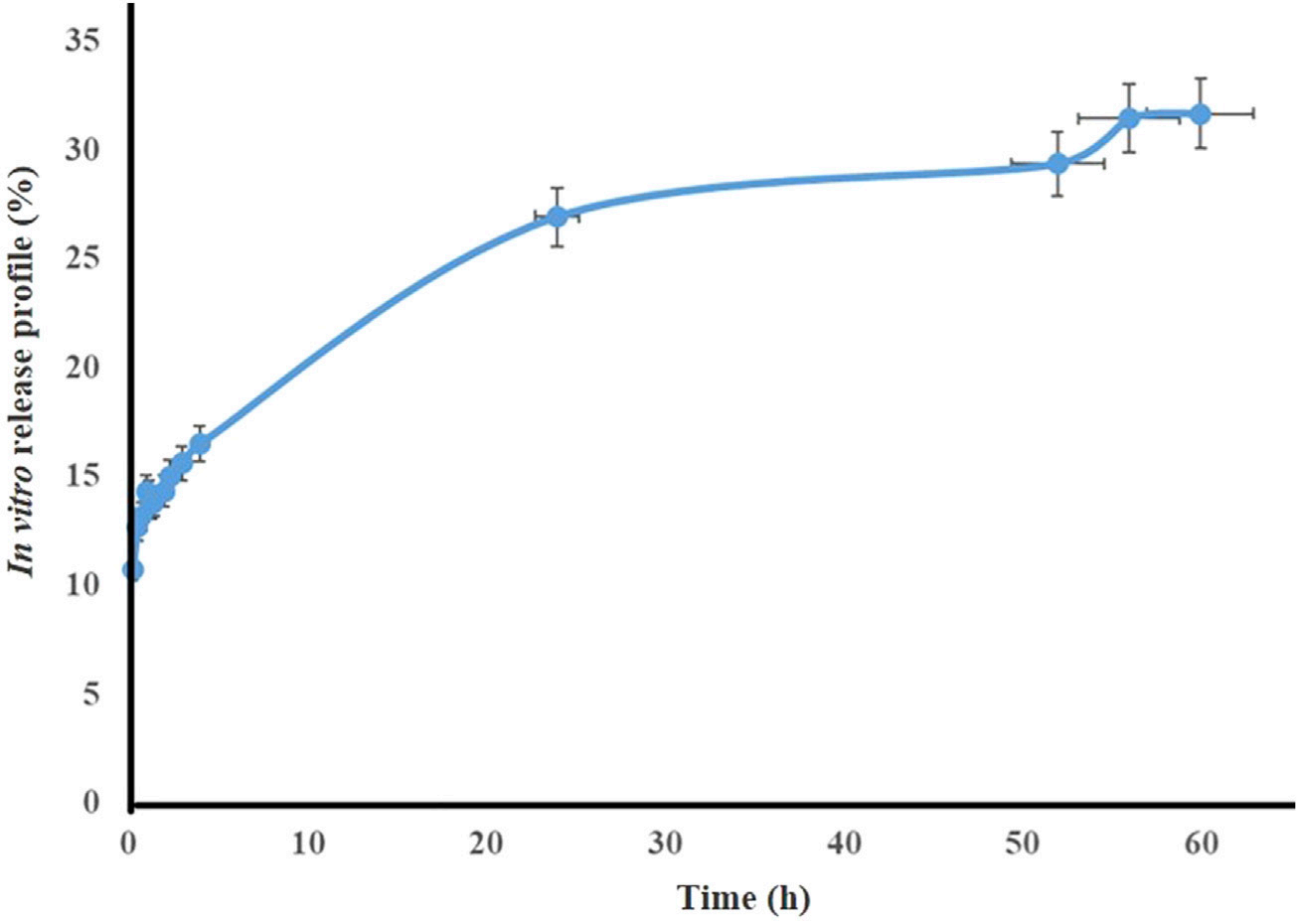
Release percentage of *TG* extract from optimal PVA/PEO/CS/*TG* nanofibers (*n* = 3).

The release profile of *TG* extract from the optimal nanofibers (PVA/PEO/CS) at pH 7.4 at different times is shown in Figure 3. The results show that the total extract release from the formulation after 60 h is close to 30%.

### 3.3 Swelling test

The swelling test was conducted three times, and the average swelling percentage of nanofibers was calculated to be 86.88%, which indicated the high ability of water absorption by the nanofibers (Table 1). This property can be very effective in absorbing secretions around the wound and keeping its surface dry. Water angle test studies on the surface of the nanofibers showed that this surface is one of the most hydrophilic surfaces, which could increase cell adhesion and facilitate the wound-healing process.

**TABLE 1:** Results of the swelling test.

Inflation (%)	W_t_ = Secondary weight (mg)	W_0_ = Initial weight (mg)
89.06	85.0	9.3
85.01	44.7	6.7
85.71	70.0	10.0
86.88	Average percentage of inflation	

### 3.4 Mechanical properties of nanofibers

**FIGURE 4:**
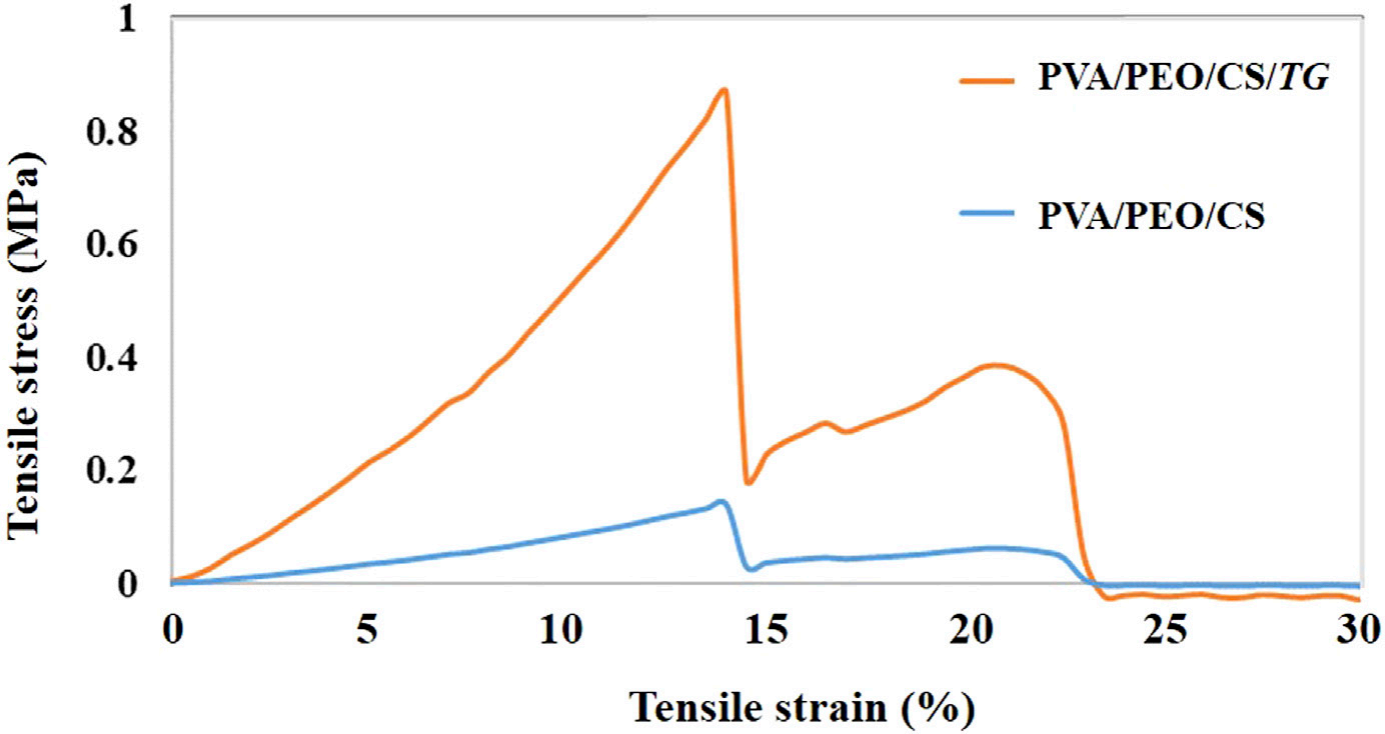
The stress-strain curve of fiber prepared from PVA/PEO/CS (polymer group) without and with *TG* (drug) extract.

The stress-strain curve of electrospun nanofibers without extract and with *TG* extract is presented in Figure 4. According to the results, the modulus of elasticity at all levels of nanofibers with extract is remarkably enhanced in comparison to nanofibers without extract. Both the modulus of elasticity and the tensile strength are higher in nanofibers with extracts than in nanofibers without extracts. Consequently, it is evident from the results that the mechanical strength of the PVA/PEO/CS nanofiber containing the *TG* extract has increased and as a result, the stress-strain curve has been transferred to higher stresses.

### 3.5 Wound contraction rate

**FIGURE 5:**
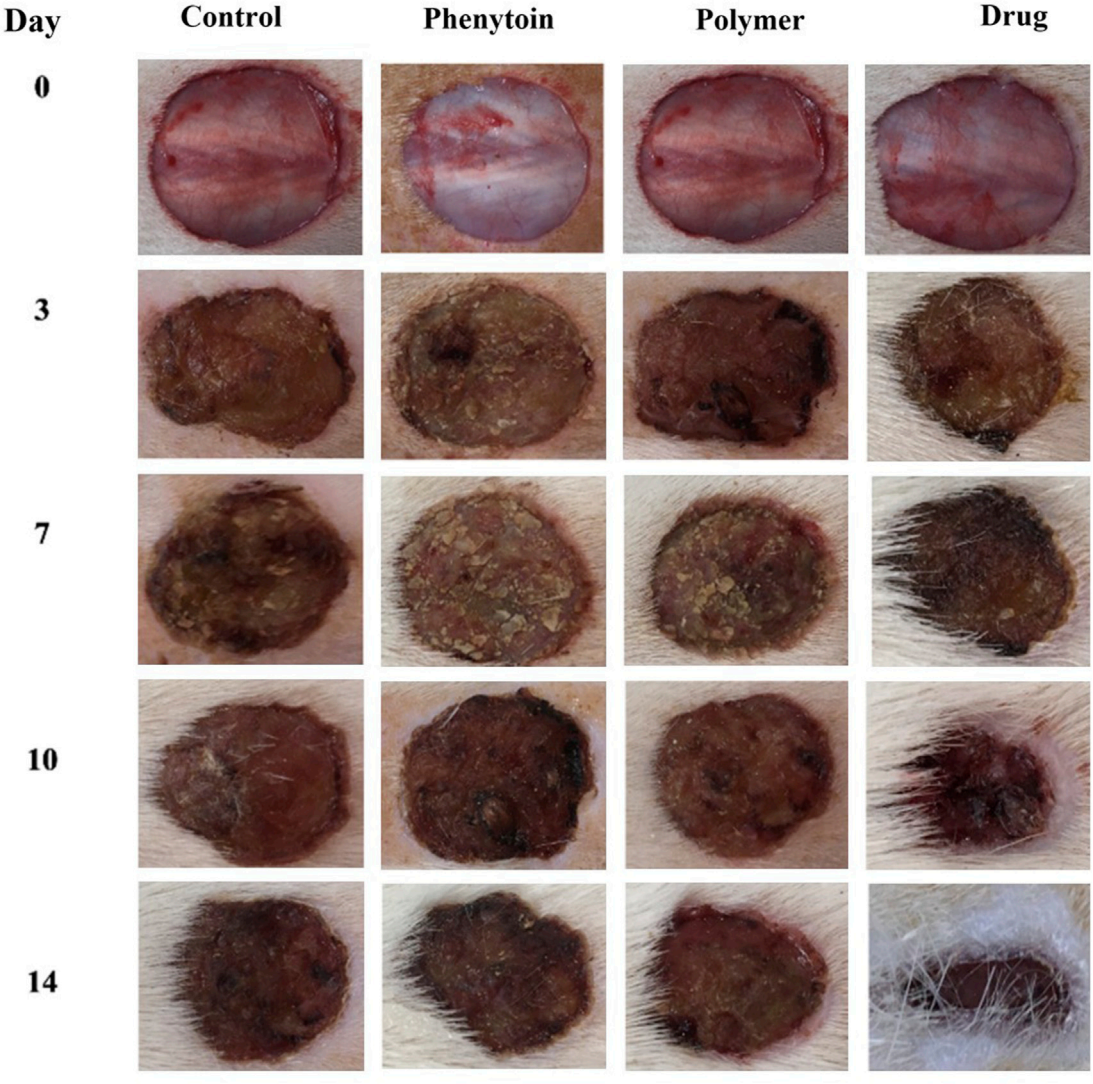
The macroscopic evaluation of wound size in normal saline, phenytoin-treated, PVA/PEO/CS, and PVA/PEO/CS/*TG* nanofibers.

**FIGURE 6:**
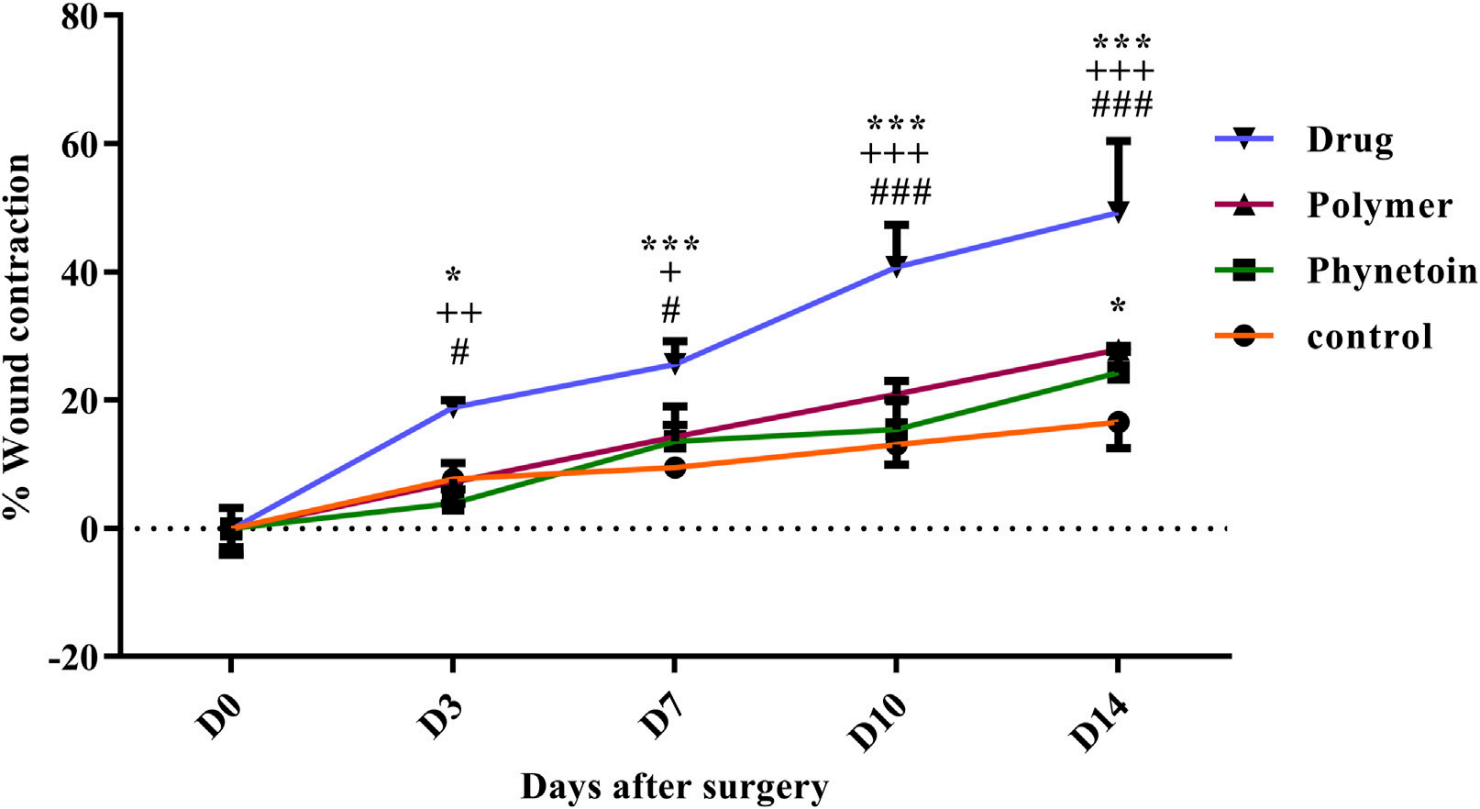
Wound closure rate of various treated groups on different days following the surgical procedure. *: *p* < 0.05, and ***: *p* < 0.001, vs. control group (normal saline); +: *p* < 0.05, ++: *p* < 0.01, and +++: *p* < 0.001, vs. phenytoin group; #: *p* < 0.05, and ###: *p* < 0.001 indicated a significant difference between polymer and drug groups.

In order to assess the efficacy of nanofibers in promoting wound healing, the size of the wounds was measured in different treated groups on days 0, 3, 7, 10, and 14. According to the results, rats whose wounds were treated with PVA/PEO/CS/*TG* nanofibers showed a significant reduction in their wound size compared to the normal saline, phenytoin, and polymer groups after day 14. Thus, the wound healing activity of PVA/PEO/CS/*TG* nanofibers was notably higher than other treated groups at all the time intervals assessed. Figure 5 represents the macroscopic trends of wound healing, and Figure 6 shows associated statistical results on wound contraction.

### 3.6 Histopathological analysis

**FIGURE 7:**
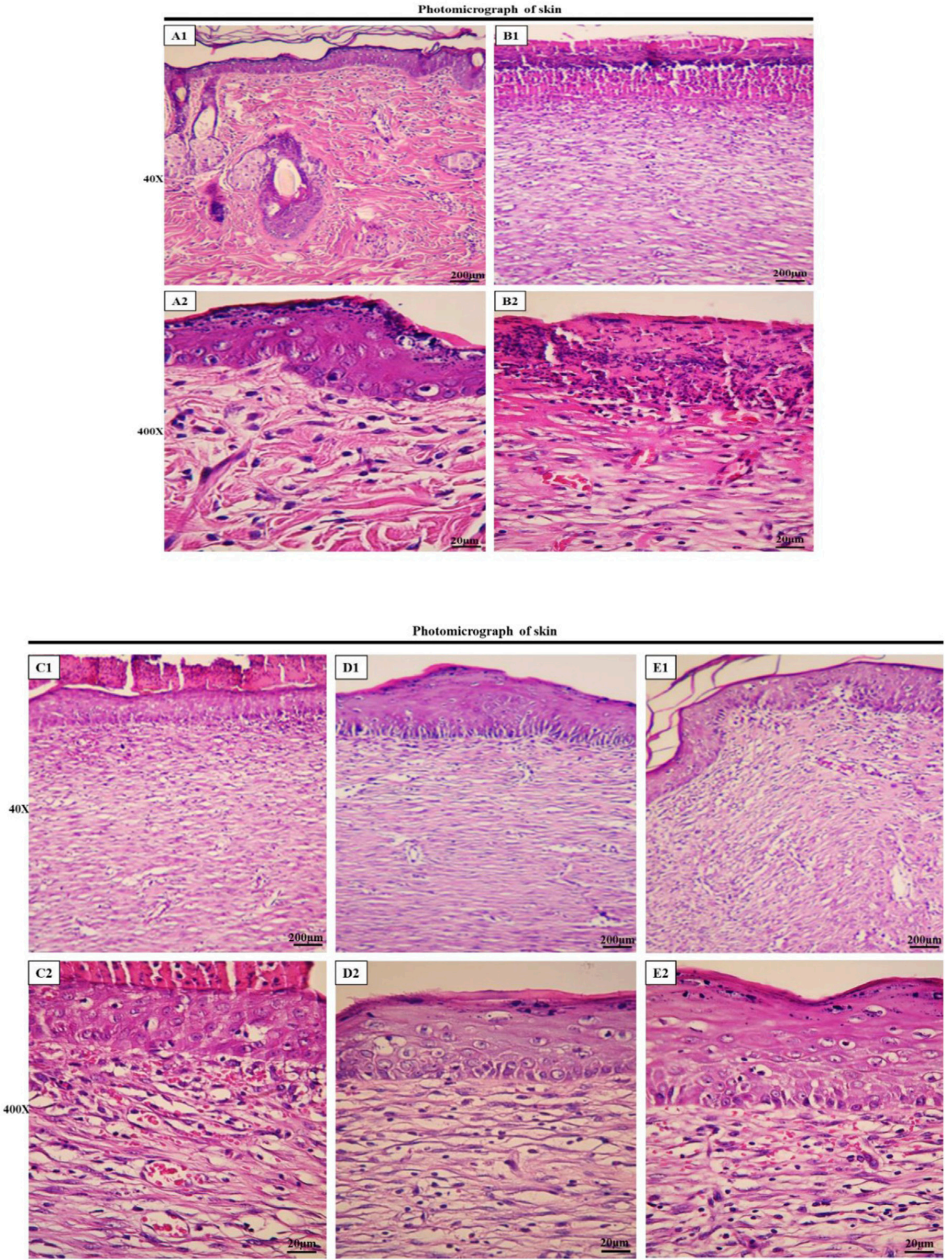
Histopathological evaluation of skin. Skin sections in the different groups (H&E; Bar = 200 µm for **(A1, B1, C1, D1, E1)**; 20 µm for **(A2, B2, C2, D2, E2)**. **(A1, A2)** ×40 and ×400: normal healthy skin is a thick tissue anatomically encompassing two main sections, namely the epidermis and dermis, which is not of main findings of the present study and presented just for comparison. The epidermis is composed of a stratified squamous epithelium that is keratinized, whereas the dermis is constituted of a diverse array of appendages such as hair follicles, adipose glands, and multiple cellular components along with organized connective tissue patterns. **(B1, B2)** ×40 and ×400: magnifications of the negative control group skin tissue sections (normal saline). **(C1, C2)** ×40, and ×400: magnifications of the PVA/PEO/CS-treated group skin tissue. **(D1, D2)** ×40, and ×400: magnifications of the PVA/PEO/CS/*TG*-treated group skin tissue. **(E1, E2)** ×40, and ×400: magnifications of the phenytoin-treated group.

Histological assessment of tissue samples from the wound area on the final day was conducted using H&E staining. Figure 7 illustrates the magnifications of tissue sections obtained from the studied groups. Additionally, normal skin tissue sections (Figures 7A1, A2) were provided for comparison, and not as main findings of the present study. The structure of healthy skin (Figures 7A1, A2) is a thick tissue, in which different parts of it, such as the epidermis and dermis, encompassing keratinized stratified squamous epithelium, fat glands, hair follicles, types of connective cells, multiple cellular components, and clusters of connective fibers are seen. In the normal saline (negative control) group, the tissue structure was disrupted, the epidermis was not formed on a large surface, and the dermis displayed an irregular and detached structure with a lack of appendages, along with a notable infiltration of inflammatory cells. In addition, in the extracellular matrix, collagen fibers are low-density and irregular and no skin appendages were observed (Figures 7B1, B2). In the PVA/PEO/CS-treated group, skin tissue structure improved and in some cases, necrotic tissue and inflammatory cell accumulations were observed (Figures 7C1, C2). However, in the groups treated with PVA/PEO/CS/*TG* nanofibers (Figures 7D1, D2) and phenytoin (positive control) (Figures 7E1, E2), the extent of skin tissue damage was reduced to some degree in comparison to the normal saline group. The wrinkled epidermis displayed significant regeneration. Moreover, the dermis represented a high density of collagen fibers from connective tissue, accompanied by the presence of normal cells. Additionally, the percentage of collagen production motility in the PVA/PEO/CS/*TG* nanofibers group was higher than both phenytoin and PVA/PEO/CS nanofibers groups.

**FIGURE 8:**
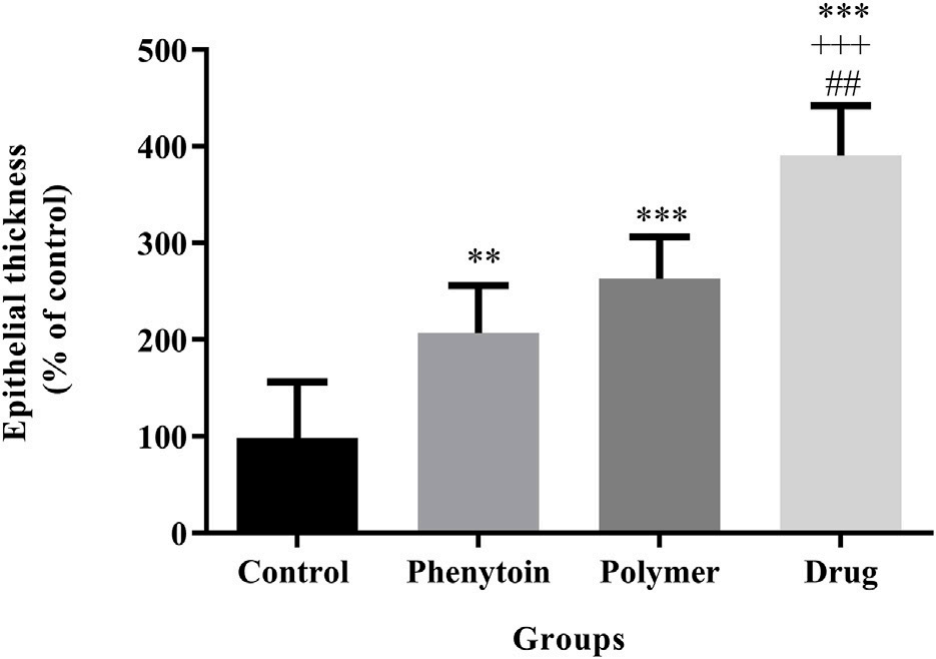
Regeneration of epithelial thickness in the studied groups. Treatment with PVA/PEO/CS/*TG* resulted in a considerable increase in epithelial thickness regeneration. **: *p* < 0.01 and ***: *p* < 0.001 vs. control group (normal saline); +++: *p* < 0.001, vs. phenytoin group; ##: *p* < 0.01 indicated a significant difference between polymer and drug groups.

**FIGURE 9:**
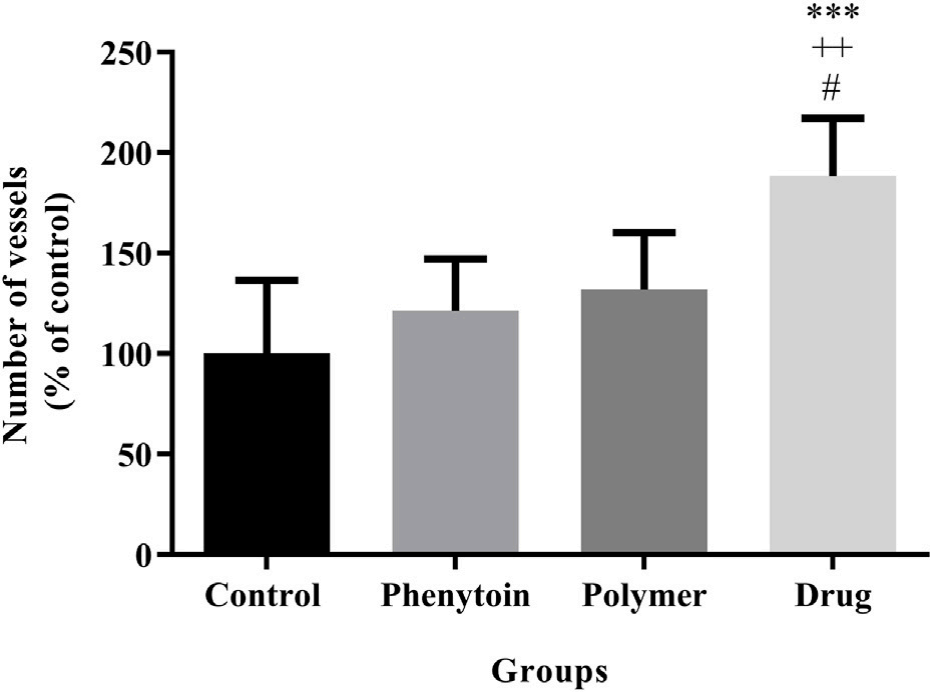
Number of blood vessels in the studied groups. Treatment with PVA/PEO/CS/*TG* resulted in a considerable increase in blood vessel number. ***: *p* < 0.001 vs. control group (normal saline); ++: *p* < 0.01, vs. phenytoin group; #: *p* < 0.05 indicated a significant difference between polymer and drug groups.

In the microscopic examination of tissue sections, the thickness of the epithelium (Figure 8) and the blood vessel number in the treated groups (Figure 9) were evaluated. Our results were indicative of a significant increase in the regeneration of epithelium thickness (epithelialization) (*p* < 0.001) and the blood vessel number (*p* < 0.001) in the group treated with PVA/PEO/CS/*TG* in comparison to the normal saline group. Moreover, PVA/PEO/CS/*TG* administration showed a remarkable elevation in comparison to the phenytoin (*p* < 0.001), and the PVA/PEO/CS nanofibers group (polymer) (*p* < 0.001) groups. The PVA/PEO/CS/TG group was also associated with an increase in the number of blood vessels compared to the other two groups (*p* < 0.05). These results showed that PVA/PEO/CS/*TG* treatment was able to accelerate the wound healing process through stimulation of angiogenesis and epithelialization.

### 3.7 Nitrite assay

**FIGURE 10:**
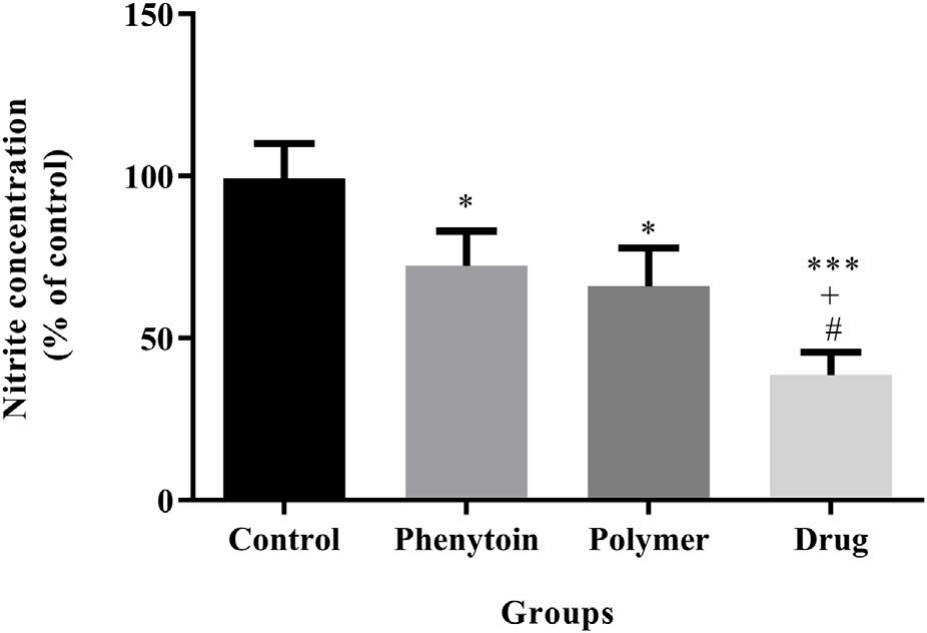
Effects of PVA/PEO/CS/*TG* on serum nitrite levels. Treatment with PVA/PEO/CS/*TG* resulted in a notable reduction in nitrite levels. *: *p* < 0.05 and ***: *p* < 0.001 vs. control group (normal saline); +: *p* < 0.05, vs. phenytoin group; #: *p* < 0.05 indicated a significant difference between polymer and drug groups.

According to the analysis illustrated in Figure 10, the serum levels of nitrite were significantly attenuated in all groups treated with 1% phenytoin cream, PVA/PEO/CS, and PVA/PEO/CS/*TG* compared to the normal saline group (*p* < 0.05).

### 3.8 Catalase assay

**FIGURE 11:**
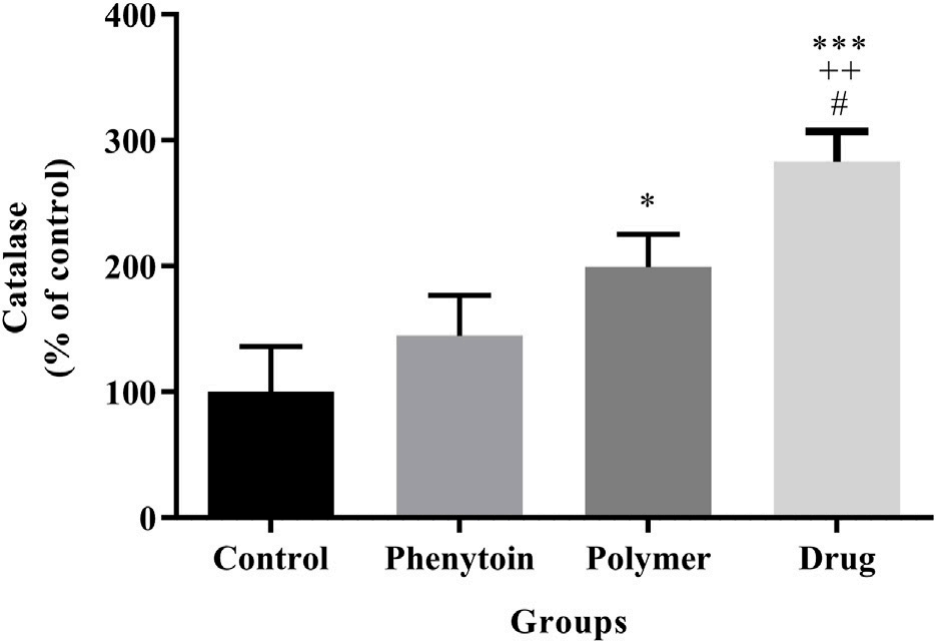
Measurement of catalase by hydrogen peroxide assay in the studied groups. Treatment with PVA/PEO/CS/*TG* exhibited a significant elevation of serum CAT levels. *: *p* < 0.05 and ***: *p* < 0.001 vs. control group (normal saline); ++: *p* < 0.01, vs. phenytoin group; #: *p* < 0.05 indicated a significant difference between polymer and drug groups.

The level of catalase in the serum is regarded as a factor with antioxidant properties, and an elevated level of catalase is deemed favorable. It was found that (Figure 11) all treatments notably elevated CAT activity compared to the normal saline group (*p* < 0.05).

## 4 Discussion

In this study, nanofibers PVA/PEO/CS (1 g PVA, 0.3 g PEO, and 1 g CS) were synthesized by the electrospinning method, and *TG* extract was loaded on the surface of nanofibers. Different concentrations of the extract were loaded on nanofibers (10, 50, and 90% w/v). Based on the results of light microscopy, the best nanofiber with 50% *TG* extract was obtained. Other concentrations of the extract led to the production of irregular and unsuitable nanofibers. Optimal nanofibers (PVA/PEO/CS) containing *TG* extract were used for wound healing in rats. High tensile strength, ease of use, and handling of materials used for wound dressing are very important features of these nanofibers. For this reason, the tensile strength of the prepared nanofiber was investigated, and based on the results, the tensile strength of this nanofiber was high and acceptable. We exhibited the wound-healing efficacy of the PVA/PEO/CS/*TG* nanofibers in a rat model of excision wound repair. Histopathological evaluation confirmed wound healing activity of PVA/PEO/CS/*TG* nanofibers, as well. Additionally, we showed the antioxidant capabilities of the PVA/PEO/CS/*TG* nanofibers through CAT and nitrite assay. According to wound closure results, PVA/PEO/CS/TG nanofibers have the potential to speed up the wound healing process in comparison to the negative control. From the mechanistic point of view, the wound-healing activity of PVA/PEO/CS/*TG* nanofibers could be attributed to the presence of diverse constituents like phenolic compounds and flavonoids that are able to speed up wound healing and exhibit antioxidant properties. Our study displayed that PVA/PEO/CS/*TG* nanofibers could reduce levels of serum nitrite and increase CAT serum levels, indicating its systemic antioxidant activity that contributes to the promotion of wound repair. According to our results, Bayrami et al. showed wound healing potential of the total extract and fractions of *TG*. They also represented luteolin as one of the active compounds responsible for wound healing by stimulating the proliferation and migration of skin fibroblasts (Bayrami et al., 2018). In another study by Heidari and colleagues, the burn wound-healing activity of the topical standardized *TG* extract was investigated. The ointment derived from the extract of aerial parts of *TG*, which had a concentration of 10%, exhibited notable advantageous impacts on an animal model of second-degree burn wounds. These effects were observed in terms of wound closure, histological analysis, and oxidative stress biomarkers (Heidari et al., 2019). Considering the antioxidant and anti-inflammatory effects of *TG* clarified by the high content of phenolic compounds of the plant, as well as the advantages of using nanofiber wound dressings, the PVA/PEO/CS/*TG* nanofibers can serve as a natural remedy for the treatment of wounds. No toxicity was found for *TG* during our previous study, so more evaluation of toxic effects was not necessary in the current report (Farzaei et al., 2013).

In the TIM, TG has been employed as a bleeding stopper and wound healer, for diverse gastrointestinal and skin disorders (Karimi, 2014; Ziafatdoost and Mirazi, 2016). Previously and in different reports, the chemical composition of *TG* was revealed. Different species of *Tragopogon* contain phenolic and flavonoid compounds (Zidorn et al., 2005). The major phenolic compounds of the plant include gallic acid, catechins, caffeic acid, and ferulic acid. The mechanism of action of these phytochemicals is based on stopping oxidative stress, lipid peroxidation, and modulating inflammatory mediators (Al-Rimawi et al., 2016). Farzaei et al. showed that *n*-hexadecanoic acid (22.0%), beta-caryophyllene (7.5%), heneicosane (6.6%), and nonanal (5.2%) are the major compounds of *TG* (Farzaei et al., 2014b), thereby indicated anti-inflammatory effects (Farzaei et al., 2015). During a bio-guided fractionation and isolation of active components from *TG*, luteolin was found to stimulate the proliferation and migration of skin fibroblast cells (Bayrami et al., 2018). Recent works also showed the healing potential of *TG* in wound burns in rats (Heidari et al., 2019). Furthermore, numerous studies have validated the pharmacological and biological effects of *TG* encompassing wound healing, antioxidant, anti-inflammatory, analgesic, and antimicrobial properties, alongside its advantageous impacts on gastrointestinal diseases (Abdalla and Zidorn, 2020).

The utilization of nanotechnology systems to administer natural compounds has the potential to yield substantial enhancements in the effectiveness of wound treatments (Vitale et al., 2022). Numerous research groups worldwide are currently conducting biomedical studies, designing, and manufacturing new wound dressings. Nanofiber structures can properly interact with skin cells and their environment and speed up the wound-healing process (Ehterami et al., 2018). Wound dressings play a crucial role in the management of wound healing. Their purpose is to protect the wound from external dangers and accelerate the healing process (Farzaei et al., 2014b; 2015; Bayrami et al., 2018; Heidari et al., 2019; Liu et al., 2021). Currently, the market offers a variety of common dressings, such as film, sponge, foam, hydrogel, and nanofiber membranes. Among these, electrospun nanofiber membranes represent a novel category of materials. These membranes are characterized by their high surface-to-volume ratio, density, remarkable versatility, suitable three-dimensional structure, and notable microporosity, allowing them to be utilized in different biomedical applications, including wound dressings, drug delivery, and tissue engineering scaffolds (Farzaei et al., 2014b; 2015; Bayrami et al., 2018; Heidari et al., 2019; Liu et al., 2021; Al-Abduljabbar and Farooq, 2022; Zulkifli et al., 2023). Nanofiber wound dressings produced through the electrospinning method offer several advantages such as high similarity of their structure and biological function to the natural extracellular matrix, which creates an optimal microenvironment for cell proliferation, differentiation, and migration (Farzaei et al., 2014b; Farzaei et al., 2015; Bayrami et al., 2018; Heidari et al., 2019; Lan et al., 2021; Liu et al., 2021; Al-Abduljabbar and Farooq, 2022; Zulkifli et al., 2023). In addition, the wide surface area and specific structure of the nanofiber membrane enable the effective loading of different biologically active ingredients, such as phytochemicals, growth factors, antibacterial drugs, herbal extracts, and vitamins (Farzaei et al., 2014b; 2015; Bayrami et al., 2018; Heidari et al., 2019; Liu et al., 2021). Therefore, designing, manufacturing, and producing advanced and smart wound dressings of *TG* with the ability for wound healing can be effective in reducing the high costs of treatment and improving the health of patients.

## 5 Conclusion

The findings of this study demonstrated the wound-healing activity of PVA/PEO/CS/*TG* nanofibers in a rat model of excision wound repair. Furthermore, we have elucidated the mechanism by which these nanofibers exert their effects, namely through their antioxidative capacity as demonstrated by the reduction in nitrite levels and elevation in catalase levels in serum samples taken from the treated rats. These significant findings contribute to a more comprehensive understanding of the wound-healing activity exhibited by the *T. graminifolius* DC. extract. However, it is imperative to conduct additional experimental and clinical studies and employ novel analytical methods to characterize major active ingredients of *TG* and validate the utilization of PVA/PEO/CS/*TG* nanofibers as a viable remedy for wound healing.

## Data Availability

The raw data supporting the conclusions of this article will be made available by the authors, without undue reservation.
