# Supplementary material for: Preparation of Tragopogon graminifolius-loaded electrospun nanofibers and evaluating its wound healing activity in a rat model of skin scar
Source: Front Pharmacol. 2025 Jan 31;16:1533010. doi: 10.3389/fphar.2025.1533010 (PMC11825791; doi:10.3389/fphar.2025.1533010)
Supplement: Supplementary file 1 [file DataSheet1.docx]

**Supporting Information file**

**FT-IR analysis**

Concisely, to investigate tensile and bending vibrations in PVA/PEO/CS and PVA/PEO/CS/*TG* nanofibers, these samples were mixed with KBr (spectroscopy grade, Sigma Aldrich) powder and compressed into tablets. The resulting mixture was then inserted into the FT-IR sample holder and compacted. Spectra were captured within the scanning range of 200–4000 cm^-1^ using a spectral resolution of 4 cm^-1^ through the FT-IR spectrophotometer (IR Prestige-21 Shimadzu Spectrometer, Kyoto, Japan).

The FT-IR spectra of PEO, CS, PVA, and optimal nanofiber containing PVA/PEO/CS with *TG* extract are shown in **Supplementary Figure 1**. In the case of PEO, the following stretching peaks were identified: C-C stretching vibrations (3386 cm^−1^), sp^2^ C‐H stretching vibrations (842.8 cm^−1^), C-O-C stretching frequency (1132.2 cm^−1^), C=C absorption band (1604.7 cm^−1^), C-O stretching frequency (1961.1 cm^−1^), C‐H stretching vibrations (2814.4 cm^−1^), CH_2_ weak stretching absorptions (2873.9 and 2927.2 cm^−1^), and finally O-H vibration (3414 cm^−1^). In addition, in the FT-IR spectrum of PEO, the bending vibrations of C-H (947.4 cm^−1^), C-O bending absorptions (1033.8, 1062.7, and 1109.6 cm^−1^), CH_2_ bending vibrations (1371.3 and 1427.3 cm^−1^), and eventually bending vibration of O-H (1591.2 cm^-1^) were characterized (Farea et al., 2020).

The main functional groups appearing in the IR spectrum of CS are as follows: bending vibrations of C-O at 993.3, 1026.13, and 1072.04 cm^-1^, stretching vibration of C-O-C at 1153.4 cm-1, bending vibration of C-N at 1217 cm^-1^, stretching vibration of C-H in 1421.5 cm^-1^, stretching vibrations of CH_2_-OH, CH-OH, and C-H in 1381, 1641.4, 2862.3 and 2916.4 cm^-1^, respectively. As the hydrogen bond is formed between the NH_2_ and the O-H groups in chitosan, the resulting stretching absorptions appear in the range of 3307.3 to 3464.1 cm^-1^ (Fernandes Queiroz et al., 2014).

In the FT-IR spectrum of PVA, the peak shown at 840.9 cm^-1^ was assigned to the symmetrical C-C stretching vibration. C-H bending vibration and C-O-C stretching vibration appeared at 947.4 and 1132.2 cm^-1^, respectively. Also, vibrations at 10333.8, 1062.7, and 1109 cm^-1^ were related to C-O bending vibrations. The peaks shown in 1371.3 and 1427.3 cm^-1^ were attributed to the bending vibrations of the CH_2_ group. The bending vibration and stretching vibration of O-H appeared in 1591.2 and 3332.9 cm^-1^, respectively. Besides, C=C and C=O stretching vibrations can also be seen in 1651.9 and 1732.0 cm^-1^, respectively (Uma Maheshwari et al., 2014).


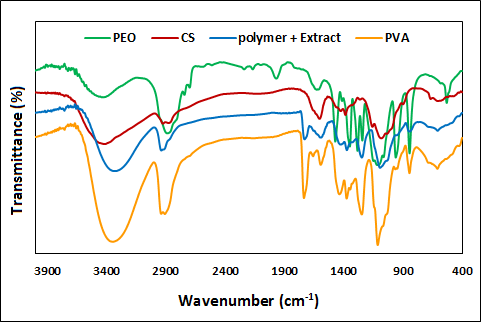


**Supplementary Figure 1.** FT-IR spectra of polymers and polymer + extract, PEO, CS, and PVA.

**References**

Farea, M. O., Abdelghany, A. M., Meikhail, M. S., and Oraby, A. H. (2020). Effect of cesium bromide on the structural, optical, thermal and electrical properties of polyvinyl alcohol and polyethylene oxide. *J. Mater. Res. Technol.* 9, 1530–1538. doi: 10.1016/j.jmrt.2019.11.078

Fernandes Queiroz, M., Melo, K., Sabry, D., Sassaki, G., and Rocha, H. (2014). Does the Use of Chitosan Contribute to Oxalate Kidney Stone Formation? *Mar. Drugs* 13, 141–158. doi: 10.3390/md13010141

Uma Maheshwari, S., Samuel, V. K., and Nagiah, N. (2014). Fabrication and evaluation of (PVA/HAp/PCL) bilayer composites as potential scaffolds for bone tissue regeneration application. *Ceram. Int.* 40, 8469–8477. doi: 10.1016/j.ceramint.2014.01.058
